# (*E*)-3-(4-Meth­oxy­phen­yl)-3-[3-(4-meth­oxy­phen­yl)-1*H*-pyrazol-1-yl]prop-2-enal

**DOI:** 10.1107/S1600536813007678

**Published:** 2013-03-28

**Authors:** V. Susindran, S. Athimoolam, S. Asath Bahadur, R. Manikannan, S. Muthusubramanian

**Affiliations:** a Department of Lighthouses & Lightships, Ministry of Shipping, Nagapattinam Lighthouse & DGPS Station, Nagapattinam 611 001, India; b Department of Physics, University College of Engineering, Nagercoil, Anna University, Tirunelveli Region, Nagercoil 629 004, India; c Department of Physics, Kalasalingam University, Anand Nagar, Krishnan Koil 626 190, India; d Institute of Organic Chemistry and Technology, Faculty of Chemical Technology, University of Pardubice, Pardubice 53210, Czech Republic; e Department of Organic Chemistry, Madurai Kamaraj University, Madurai 625 021, India

## Abstract

In the title mol­ecule, C_20_H_18_N_2_O_3_, the pyrazole ring forms a dihedral angle of 2.2 (1)° with its meth­oxy­phenyl substituent and a dihedral angle of 67.2 (1)° with the benzene substituent on the propenal unit. In the crystal, mol­ecules are connected by weak C—H⋯O hydrogen bonds, forming *R*_2_^2^(26) and *R*_2_^2^(28) cyclic dimers that lie about crystallographic inversion centres. These dimers are further linked through C—H⋯O and C—H⋯N hydrogen bonds, forming *C*(8), *C*(9), *C*(10) and *C*(16) chain motifs. These primary motifs are further linked to form secondary *C*_2_^2^(15) chains and *R*_2_^2^(18) rings.

## Related literature

For the pharmacological and medicinal properties of pyrazole compounds, see: Baraldi *et al.* (1998 ▶); Bruno *et al.* (1990 ▶); Chen & Li (1998 ▶); Cottineau *et al.* (2002 ▶); Londershausen (1996 ▶); Mishra *et al.* (1998 ▶); Smith *et al.* (2001 ▶). For related structures, see: Susindran *et al.* (2010*a* ▶,*b* ▶, 2012 ▶). For hydrogen-bond motifs, see: Etter *et al.* (1990 ▶).
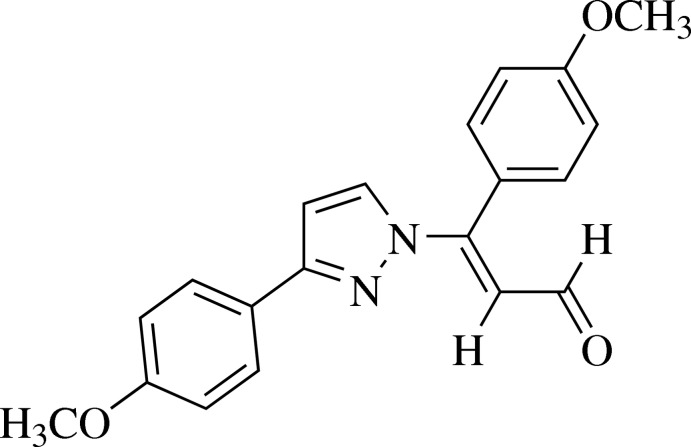


## Experimental

### 

#### Crystal data

C_20_H_18_N_2_O_3_
*M* *_r_* = 334.36
Triclinic, 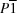

*a* = 8.8081 (6) Å
*b* = 9.8474 (5) Å
*c* = 10.3292 (8) Å
α = 94.997 (12)°
β = 93.811 (14)°
γ = 106.719 (13)°
*V* = 850.85 (10) Å^3^
*Z* = 2
Mo *K*α radiation
μ = 0.09 mm^−1^
*T* = 293 K
0.22 × 0.19 × 0.15 mm

#### Data collection

Bruker SMART APEX CCD diffractometer
8253 measured reflections
2993 independent reflections
2677 reflections with *I* > 2σ(*I*)
*R*_int_ = 0.020

#### Refinement

*R*[*F* ^2^ > 2σ(*F* ^2^)] = 0.040
*wR*(*F* ^2^) = 0.107
*S* = 1.04
2993 reflections
228 parameters
H-atom parameters constrained
Δρ_max_ = 0.14 e Å^−3^
Δρ_min_ = −0.25 e Å^−3^

### 

Data collection: *SMART* (Bruker, 2001 ▶); cell refinement: *SAINT* (Bruker, 2001 ▶); data reduction: *SAINT*; program(s) used to solve structure: *SHELXTL* (Sheldrick, 2008 ▶); program(s) used to refine structure: *SHELXTL*; molecular graphics: *PLATON* (Spek, 2009 ▶); software used to prepare material for publication: *SHELXTL*.

## Supplementary Material

Crystal structure: contains datablock(s) global, I. DOI: 10.1107/S1600536813007678/sj5307sup1.cif

Structure factors: contains datablock(s) I. DOI: 10.1107/S1600536813007678/sj5307Isup2.hkl

Supplementary material file. DOI: 10.1107/S1600536813007678/sj5307Isup3.cml

Additional supplementary materials:  crystallographic information; 3D view; checkCIF report

## Figures and Tables

**Table 1:** Hydrogen-bond geometry (Å, °)

*D*—H⋯*A*	*D*—H	H⋯*A*	*D*⋯*A*	*D*—H⋯*A*
C5—H5⋯O3^i^	0.93	2.73	3.366 (2)	126
C18—H18*C*⋯N2^i^	0.96	2.74	3.627 (2)	155
C14—H14⋯O2^ii^	0.93	2.49	3.301 (2)	145
C33—H33⋯O1^iii^	0.93	2.82	3.642 (2)	148
C37—H37*B*⋯O2^iv^	0.96	2.75	3.688 (2)	166
C37—H37*C*⋯O1^v^	0.96	2.76	3.650 (2)	155

Symmetry codes: (i) 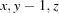; (ii) 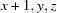; (iii) 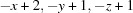; (iv) 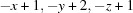; (v) 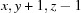.

**Fig. 1.**
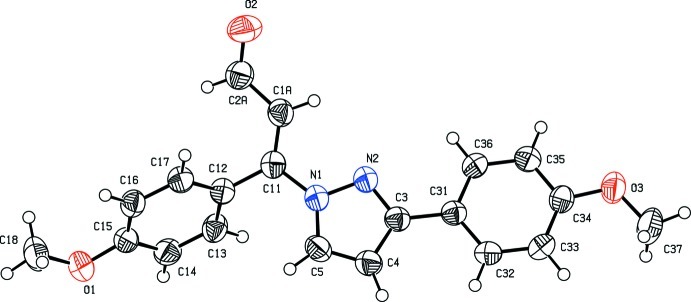
The molecular structure of the title compound with 50% probability displacement ellipsoids.

**Fig. 2.**
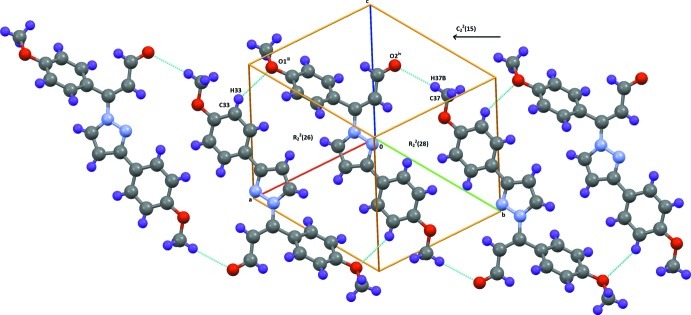
A view of the primary R22(26) and R22(28) rings and the secondary C22(15) chains.

**Fig. 3.**
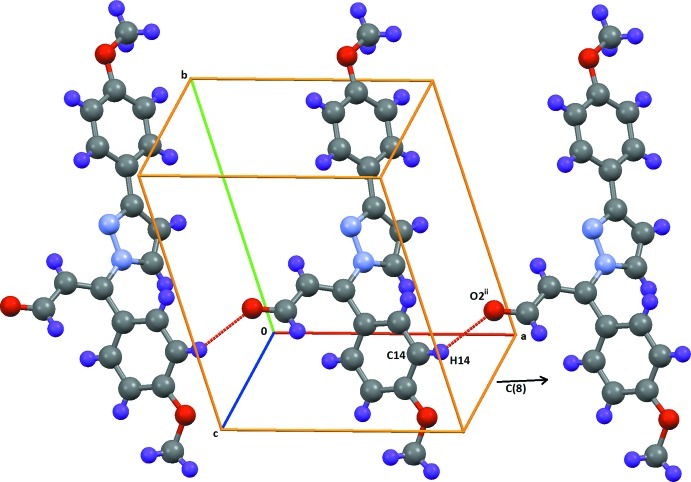
A view of the C(8) chain extending along the a-axis.

**Fig. 4.**
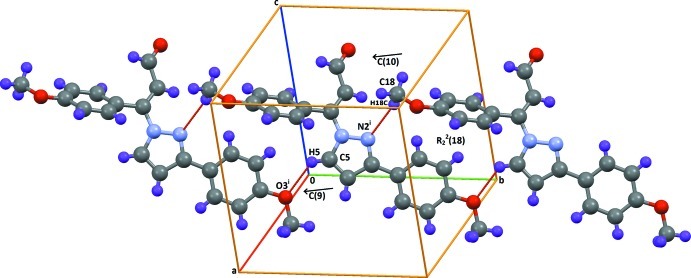
A view of the primary C(9) and C(10) chains leading to a secondary R22(18) ring motif.

**Fig. 5.**
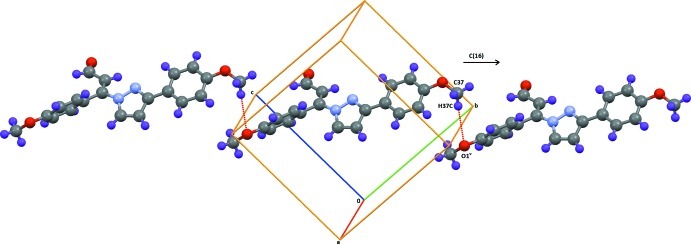
A view of a C(16) chain motif connecting the molecules in a head-to-tail fashion along the diagonal of the bc-plane.

## supplementary crystallographic information

### Comment

Pyrazoles are classified as both aromatic ring compounds and heterocyclic compounds and are characterized by a 5-membered ring structure composed of three carbon atoms and two nitrogen atoms in adjacent positions in the unsubstituted parent compound. Being so composed and having pharmacological effects on humans, they are classified as alkaloids although they are rare in nature. Pyrazole and its derivatives have been successfully tested for their antifungal (Chen & Li, 1998), antihistaminic (Mishra *et al.*,1998), anti-inflammatory (Smith *et al.*, 2001), antiarrhythmic and sedative (Bruno *et al.*, 1990), hypoglycemic (Cottineau *et al.*, 2002), antiviral (Baraldi *et al.*, 1998) and pesticidal (Londershausen, 1996) activities. Based on the above specifics and also as part of our continuing work on pyrazole related compounds (Susindran *et al.*, 2010*a*,*b*, 2012), we report here the structure of the title pyrazole derivative.

The molecular structure of the title compound is shown in Fig. 1. The phenyl rings of the methoxyphenyl groups and the plane of the pyrazole ring form dihedral angles of 2.2 (1)° (with the C31—C36 ring) and 67.2 (1)° (with the C12—C17 ring). The crystal packing is stabilized through weak intermolecular C—H···O and C—H···N interactions (Table 1). Molecules are connected around inversion centres of the unit cell making *R*_2_^2^(26) and *R*_2_^2^(28) ring motifs (Etter *et al.*, 1990) through C33—H33···O1 and C37—H37B···O2 interactions, respectively. These pairs of dimeric rings are further connected in a head-to-tail fashion by C37—H37B···O2 and C33—H33···O1 contacts, Table 1, to generate sheets of dimers approximately parallel to (112). These contacts also generate C_2_^2^(15) chains in the *ab*-plane, Fig 2.

Additional chains are generated in the crystal as follows. A C(8) chain forms along the *a*-axis through a C14—H14···O2 interaction (Fig. 3) while C5—H5···O3 and C18—H18C···N2 contacts generate C(9) and C(10) chains along *b*. A combination of these contacts also generate an *R*_2_^2^(18) ring motif (Fig. 4). Finally a C(16) chain of molecules linked in a head to tail fashion forms along the *bc* diagonal through a C37—H37C···O1 contact, Fig 5.

### Experimental

Phosphorous oxychloride (0.024 mole) was added dropwise over 5 to 10 minutes to a mixture of 1-(4-methoxyphenyl)-1-ethanone *N*-[(*E*)-1-(4-methoxyphenyl)ethylidene]hydrazone (0.003 mole) and 3 ml of dimethyl formamide cooled in ice to 0°C. The reaction mixture was then irradiated with microwaves for 30 sec. The progress of the reaction was monitored by TLC. After completion of the reaction, the reaction mixture was poured into crushed ice and extracted with dichloromethane. The organic layer was dried over anhydrous sodium sulfate. The products were separated by column chromatography using petroleum ether and ethyl acetate mixture (98/2 v/v) as eluent. The title compound was crystallized from dichloromethane.

### Refinement

All the H atoms were positioned geometrically and refined using a riding model, with C—H = 0.93 - 0.96 Å and *U*_iso_(H) = 1.2 - 1.5 *U*_eq_ (parent atom).

### Crystal data

**Table:**

C_20_H_18_N_2_O_3_	*Z* = 2
*M**_r_* = 334.36	*F*(000) = 352
Triclinic, *P*1	*D*_x_ = 1.305 Mg m^−^^3^
Hall symbol: -P 1	Mo *K*α radiation, λ = 0.71073 Å
*a* = 8.8081 (6) Å	Cell parameters from 3011 reflections
*b* = 9.8474 (5) Å	θ = 2.2–24.3°
*c* = 10.3292 (8) Å	µ = 0.09 mm^−^^1^
α = 94.997 (12)°	*T* = 293 K
β = 93.811 (14)°	Block, colourless
γ = 106.719 (13)°	0.22 × 0.19 × 0.15 mm
*V* = 850.85 (10) Å^3^	

### Data collection

**Table:**

Bruker SMART APEX CCD diffractometer	2677 reflections with *I* > 2σ(*I*)
Radiation source: fine-focus sealed tube	*R*_int_ = 0.020
Graphite monochromator	θ_max_ = 25.0°, θ_min_ = 2.0°
ω scans	*h* = −10→10
8253 measured reflections	*k* = −11→11
2993 independent reflections	*l* = −12→12

### Refinement

**Table:**

Refinement on *F*^2^	Primary atom site location: structure-invariant direct methods
Least-squares matrix: full	Secondary atom site location: difference Fourier map
*R*[*F*^2^ > 2σ(*F*^2^)] = 0.040	Hydrogen site location: inferred from neighbouring sites
*wR*(*F*^2^) = 0.107	H-atom parameters constrained
*S* = 1.04	*w* = 1/[σ^2^(*F*_o_^2^) + (0.057*P*)^2^ + 0.1393*P*] where *P* = (*F*_o_^2^ + 2*F*_c_^2^)/3
2993 reflections	(Δ/σ)_max_ < 0.001
228 parameters	Δρ_max_ = 0.14 e Å^−^^3^
0 restraints	Δρ_min_ = −0.25 e Å^−^^3^

### Special details

**Table:**

Geometry. All e.s.d.'s (except the e.s.d. in the dihedral angle between two l.s. planes) are estimated using the full covariance matrix. The cell e.s.d.'s are taken into account individually in the estimation of e.s.d.'s in distances, angles and torsion angles; correlations between e.s.d.'s in cell parameters are only used when they are defined by crystal symmetry. An approximate (isotropic) treatment of cell e.s.d.'s is used for estimating e.s.d.'s involving l.s. planes.
Refinement. Refinement of *F*^2^ against ALL reflections. The weighted *R*-factor *wR* and goodness of fit *S* are based on *F*^2^, conventional *R*-factors *R* are based on *F*, with *F* set to zero for negative *F*^2^. The threshold expression of *F*^2^ > σ(*F*^2^) is used only for calculating *R*-factors(gt) *etc*. and is not relevant to the choice of reflections for refinement. *R*-factors based on *F*^2^ are statistically about twice as large as those based on *F*, and *R*- factors based on ALL data will be even larger.

### Fractional atomic coordinates and isotropic or equivalent isotropic displacement parameters (Å^2^)

**Table:**

	*x*	*y*	*z*	*U*_iso_*/*U*_eq_	
N1	0.69002 (13)	0.52763 (11)	0.64078 (11)	0.0457 (3)	
N2	0.67831 (13)	0.65931 (12)	0.61936 (11)	0.0462 (3)	
C3	0.76448 (15)	0.69626 (14)	0.52034 (12)	0.0422 (3)	
C4	0.83041 (17)	0.58717 (15)	0.47685 (14)	0.0509 (3)	
H4	0.8947	0.5874	0.4089	0.061*	
C5	0.78079 (17)	0.48282 (15)	0.55426 (14)	0.0505 (3)	
H5	0.8041	0.3963	0.5496	0.061*	
C11	0.60073 (15)	0.45024 (14)	0.73278 (13)	0.0444 (3)	
C12	0.66018 (15)	0.33536 (14)	0.77906 (12)	0.0436 (3)	
C13	0.81934 (16)	0.36217 (15)	0.82620 (14)	0.0508 (3)	
H13	0.8894	0.4532	0.8269	0.061*	
C14	0.87420 (17)	0.25667 (16)	0.87148 (14)	0.0527 (4)	
H14	0.9807	0.2768	0.9028	0.063*	
C15	0.77221 (17)	0.12014 (15)	0.87087 (13)	0.0473 (3)	
C16	0.61388 (17)	0.09080 (14)	0.82448 (13)	0.0478 (3)	
H16	0.5444	−0.0005	0.8239	0.057*	
C17	0.55946 (16)	0.19790 (14)	0.77898 (13)	0.0454 (3)	
H17	0.4530	0.1774	0.7476	0.055*	
O1	0.84005 (13)	0.02394 (12)	0.91793 (11)	0.0643 (3)	
C18	0.7407 (2)	−0.11630 (18)	0.92744 (19)	0.0734 (5)	
H18A	0.6562	−0.1113	0.9801	0.110*	
H18B	0.8025	−0.1704	0.9669	0.110*	
H18C	0.6963	−0.1619	0.8417	0.110*	
C31	0.77884 (15)	0.83377 (14)	0.47012 (12)	0.0413 (3)	
C32	0.86613 (15)	0.87520 (15)	0.36586 (13)	0.0460 (3)	
H32	0.9203	0.8156	0.3287	0.055*	
C33	0.87491 (16)	1.00260 (15)	0.31559 (13)	0.0483 (3)	
H33	0.9338	1.0277	0.2453	0.058*	
C34	0.79545 (16)	1.09256 (14)	0.37054 (13)	0.0459 (3)	
C35	0.71081 (17)	1.05416 (15)	0.47681 (14)	0.0507 (3)	
H35	0.6596	1.1153	0.5157	0.061*	
C36	0.70186 (17)	0.92713 (15)	0.52510 (13)	0.0481 (3)	
H36	0.6434	0.9027	0.5958	0.058*	
O3	0.79160 (14)	1.21895 (11)	0.32819 (11)	0.0639 (3)	
C37	0.8698 (3)	1.26075 (19)	0.21679 (18)	0.0768 (5)	
H37A	0.9816	1.2730	0.2343	0.115*	
H37B	0.8542	1.3492	0.1958	0.115*	
H37C	0.8267	1.1885	0.1445	0.115*	
C1A	0.47167 (16)	0.48460 (15)	0.77072 (14)	0.0496 (3)	
H1A	0.4399	0.5534	0.7292	0.059*	
C2A	0.38042 (18)	0.42069 (15)	0.87178 (15)	0.0547 (4)	
H2A	0.4199	0.3611	0.9206	0.066*	
O2	0.25512 (13)	0.43970 (13)	0.89739 (13)	0.0758 (4)	

### Atomic displacement parameters (Å^2^)

**Table:**

	*U*^11^	*U*^22^	*U*^33^	*U*^12^	*U*^13^	*U*^23^
N1	0.0500 (6)	0.0417 (6)	0.0481 (6)	0.0163 (5)	0.0060 (5)	0.0086 (5)
N2	0.0514 (7)	0.0421 (6)	0.0482 (6)	0.0168 (5)	0.0077 (5)	0.0083 (5)
C3	0.0397 (7)	0.0464 (7)	0.0407 (7)	0.0139 (6)	0.0013 (5)	0.0043 (5)
C4	0.0551 (8)	0.0560 (8)	0.0481 (8)	0.0247 (7)	0.0105 (6)	0.0081 (6)
C5	0.0555 (8)	0.0478 (8)	0.0539 (8)	0.0242 (7)	0.0057 (6)	0.0050 (6)
C11	0.0437 (7)	0.0409 (7)	0.0446 (7)	0.0078 (6)	−0.0012 (6)	0.0040 (5)
C12	0.0432 (7)	0.0429 (7)	0.0438 (7)	0.0113 (6)	0.0027 (5)	0.0063 (5)
C13	0.0435 (7)	0.0482 (8)	0.0562 (8)	0.0048 (6)	0.0026 (6)	0.0134 (6)
C14	0.0402 (7)	0.0648 (9)	0.0546 (8)	0.0156 (7)	0.0035 (6)	0.0161 (7)
C15	0.0532 (8)	0.0531 (8)	0.0421 (7)	0.0227 (7)	0.0104 (6)	0.0121 (6)
C16	0.0518 (8)	0.0414 (7)	0.0487 (8)	0.0102 (6)	0.0072 (6)	0.0073 (6)
C17	0.0405 (7)	0.0446 (7)	0.0486 (7)	0.0097 (6)	0.0006 (6)	0.0044 (6)
O1	0.0669 (7)	0.0648 (7)	0.0729 (7)	0.0323 (6)	0.0096 (5)	0.0252 (5)
C18	0.0972 (13)	0.0589 (10)	0.0768 (12)	0.0368 (9)	0.0141 (10)	0.0253 (8)
C31	0.0380 (7)	0.0453 (7)	0.0402 (7)	0.0125 (6)	0.0012 (5)	0.0039 (5)
C32	0.0419 (7)	0.0494 (8)	0.0482 (7)	0.0156 (6)	0.0083 (6)	0.0026 (6)
C33	0.0455 (7)	0.0510 (8)	0.0455 (7)	0.0075 (6)	0.0116 (6)	0.0073 (6)
C34	0.0462 (7)	0.0405 (7)	0.0473 (7)	0.0071 (6)	0.0030 (6)	0.0053 (6)
C35	0.0560 (8)	0.0470 (8)	0.0531 (8)	0.0196 (6)	0.0136 (6)	0.0055 (6)
C36	0.0525 (8)	0.0518 (8)	0.0437 (7)	0.0176 (6)	0.0144 (6)	0.0106 (6)
O3	0.0835 (8)	0.0468 (6)	0.0661 (7)	0.0200 (5)	0.0224 (6)	0.0182 (5)
C37	0.1060 (14)	0.0580 (10)	0.0669 (11)	0.0169 (9)	0.0234 (10)	0.0238 (8)
C1A	0.0458 (8)	0.0456 (8)	0.0565 (8)	0.0119 (6)	0.0019 (6)	0.0090 (6)
C2A	0.0505 (8)	0.0477 (8)	0.0621 (9)	0.0086 (6)	0.0062 (7)	0.0046 (7)
O2	0.0561 (7)	0.0799 (8)	0.0946 (9)	0.0200 (6)	0.0264 (6)	0.0125 (7)

### Geometric parameters (Å, º)

**Table:**

N1—C5	1.3646 (18)	C18—H18A	0.9600
N1—N2	1.3656 (15)	C18—H18B	0.9600
N1—C11	1.4082 (17)	C18—H18C	0.9600
N2—C3	1.3281 (17)	C31—C32	1.3869 (18)
C3—C4	1.4151 (19)	C31—C36	1.3951 (19)
C3—C31	1.4668 (18)	C32—C33	1.3830 (19)
C4—C5	1.348 (2)	C32—H32	0.9300
C4—H4	0.9300	C33—C34	1.3852 (19)
C5—H5	0.9300	C33—H33	0.9300
C11—C1A	1.3482 (19)	C34—O3	1.3633 (16)
C11—C12	1.4779 (18)	C34—C35	1.3865 (19)
C12—C17	1.3885 (18)	C35—C36	1.3701 (19)
C12—C13	1.3950 (19)	C35—H35	0.9300
C13—C14	1.3693 (19)	C36—H36	0.9300
C13—H13	0.9300	O3—C37	1.4116 (19)
C14—C15	1.385 (2)	C37—H37A	0.9600
C14—H14	0.9300	C37—H37B	0.9600
C15—O1	1.3631 (16)	C37—H37C	0.9600
C15—C16	1.383 (2)	C1A—C2A	1.438 (2)
C16—C17	1.3821 (18)	C1A—H1A	0.9300
C16—H16	0.9300	C2A—O2	1.2133 (18)
C17—H17	0.9300	C2A—H2A	0.9300
O1—C18	1.422 (2)		
			
C5—N1—N2	111.08 (11)	O1—C18—H18B	109.5
C5—N1—C11	128.12 (11)	H18A—C18—H18B	109.5
N2—N1—C11	120.42 (11)	O1—C18—H18C	109.5
C3—N2—N1	105.15 (10)	H18A—C18—H18C	109.5
N2—C3—C4	110.69 (12)	H18B—C18—H18C	109.5
N2—C3—C31	120.28 (12)	C32—C31—C36	117.49 (12)
C4—C3—C31	129.03 (12)	C32—C31—C3	121.90 (12)
C5—C4—C3	105.76 (12)	C36—C31—C3	120.60 (12)
C5—C4—H4	127.1	C33—C32—C31	121.85 (13)
C3—C4—H4	127.1	C33—C32—H32	119.1
C4—C5—N1	107.31 (12)	C31—C32—H32	119.1
C4—C5—H5	126.3	C32—C33—C34	119.60 (12)
N1—C5—H5	126.3	C32—C33—H33	120.2
C1A—C11—N1	119.20 (12)	C34—C33—H33	120.2
C1A—C11—C12	125.76 (12)	O3—C34—C33	125.26 (12)
N1—C11—C12	115.04 (11)	O3—C34—C35	115.54 (12)
C17—C12—C13	117.75 (12)	C33—C34—C35	119.20 (13)
C17—C12—C11	121.30 (12)	C36—C35—C34	120.67 (13)
C13—C12—C11	120.94 (12)	C36—C35—H35	119.7
C14—C13—C12	121.12 (13)	C34—C35—H35	119.7
C14—C13—H13	119.4	C35—C36—C31	121.16 (13)
C12—C13—H13	119.4	C35—C36—H36	119.4
C13—C14—C15	120.43 (13)	C31—C36—H36	119.4
C13—C14—H14	119.8	C34—O3—C37	118.14 (12)
C15—C14—H14	119.8	O3—C37—H37A	109.5
O1—C15—C16	125.18 (13)	O3—C37—H37B	109.5
O1—C15—C14	115.27 (13)	H37A—C37—H37B	109.5
C16—C15—C14	119.55 (13)	O3—C37—H37C	109.5
C17—C16—C15	119.65 (13)	H37A—C37—H37C	109.5
C17—C16—H16	120.2	H37B—C37—H37C	109.5
C15—C16—H16	120.2	C11—C1A—C2A	123.61 (13)
C16—C17—C12	121.50 (12)	C11—C1A—H1A	118.2
C16—C17—H17	119.3	C2A—C1A—H1A	118.2
C12—C17—H17	119.3	O2—C2A—C1A	124.07 (15)
C15—O1—C18	118.42 (13)	O2—C2A—H2A	118.0
O1—C18—H18A	109.5	C1A—C2A—H2A	118.0
			
C5—N1—N2—C3	−0.92 (14)	C15—C16—C17—C12	−0.2 (2)
C11—N1—N2—C3	−174.43 (11)	C13—C12—C17—C16	0.3 (2)
N1—N2—C3—C4	0.69 (15)	C11—C12—C17—C16	−178.75 (12)
N1—N2—C3—C31	179.94 (11)	C16—C15—O1—C18	−3.6 (2)
N2—C3—C4—C5	−0.22 (16)	C14—C15—O1—C18	176.38 (14)
C31—C3—C4—C5	−179.40 (13)	N2—C3—C31—C32	−179.19 (12)
C3—C4—C5—N1	−0.35 (16)	C4—C3—C31—C32	−0.1 (2)
N2—N1—C5—C4	0.80 (16)	N2—C3—C31—C36	−0.28 (19)
C11—N1—C5—C4	173.69 (12)	C4—C3—C31—C36	178.82 (13)
C5—N1—C11—C1A	−152.78 (14)	C36—C31—C32—C33	−1.3 (2)
N2—N1—C11—C1A	19.52 (18)	C3—C31—C32—C33	177.64 (12)
C5—N1—C11—C12	27.04 (19)	C31—C32—C33—C34	0.3 (2)
N2—N1—C11—C12	−160.66 (11)	C32—C33—C34—O3	−178.35 (12)
C1A—C11—C12—C17	50.16 (19)	C32—C33—C34—C35	1.2 (2)
N1—C11—C12—C17	−129.64 (13)	O3—C34—C35—C36	177.84 (13)
C1A—C11—C12—C13	−128.87 (16)	C33—C34—C35—C36	−1.8 (2)
N1—C11—C12—C13	51.32 (17)	C34—C35—C36—C31	0.8 (2)
C17—C12—C13—C14	−0.3 (2)	C32—C31—C36—C35	0.7 (2)
C11—C12—C13—C14	178.74 (13)	C3—C31—C36—C35	−178.22 (12)
C12—C13—C14—C15	0.3 (2)	C33—C34—O3—C37	2.3 (2)
C13—C14—C15—O1	179.86 (13)	C35—C34—O3—C37	−177.29 (14)
C13—C14—C15—C16	−0.2 (2)	N1—C11—C1A—C2A	−174.57 (12)
O1—C15—C16—C17	−179.88 (12)	C12—C11—C1A—C2A	5.6 (2)
C14—C15—C16—C17	0.2 (2)	C11—C1A—C2A—O2	−172.01 (15)

### Hydrogen-bond geometry (Å, º)

**Table:**

*D*—H···*A*	*D*—H	H···*A*	*D*···*A*	*D*—H···*A*
C5—H5···O3^i^	0.93	2.73	3.366 (2)	126
C18—H18*C*···N2^i^	0.96	2.74	3.627 (2)	155
C14—H14···O2^ii^	0.93	2.49	3.301 (2)	145
C33—H33···O1^iii^	0.93	2.82	3.642 (2)	148
C37—H37*B*···O2^iv^	0.96	2.75	3.688 (2)	166
C37—H37*C*···O1^v^	0.96	2.76	3.650 (2)	155

Symmetry codes: (i) *x*, *y*−1, *z*; (ii) *x*+1, *y*, *z*; (iii) −*x*+2, −*y*+1, −*z*+1; (iv) −*x*+1, −*y*+2, −*z*+1; (v) *x*, *y*+1, *z*−1.
